# Protective efficacy of inactivated FHV-1 vaccine in cats following challenge with the Chinese field strains

**DOI:** 10.3389/fvets.2025.1571409

**Published:** 2025-04-24

**Authors:** Yujie Jiang, Zhiying Lai, Lingling Dai, Yuan Deng, Lintao Zhong, Shoujun Li, Gang Lu

**Affiliations:** College of Veterinary Medicine, South China Agricultural University, Guangzhou, China

**Keywords:** feline herpesvirus type 1, vaccine, immunogenicity, infectious disease, feline viral rhinotracheitis

## Abstract

Feline herpesvirus-1 (FHV-1) is a leading cause of feline viral rhinotracheitis (FVR), which mainly presents upper respiratory tract symptoms. Vaccination is the most effective strategy for controlling FHV-1. Prior to the initiation of this study, China does not have domestically produced commercially available FHV-1 vaccines using field strain as antigenic component and most corresponding imported vaccines contained feline viral rhinotracheitis, calicivirus, and panleukopenia (FVRCP) antigens. However, the protective efficacy of these vaccines against the prevalent FHV-1 strains in China remains unclear. In the present study, a total of 12 cats were randomly divided into 3 groups, which were vaccinated with FHV-1 field vaccine (Group 1 [an inactivated vaccine developed by ourselves using the Chinese field strain FHV-1 2020GD02]) and FVRCP vaccine (Group 2) and PBS (Group 3) as control, respectively. These animals received two vaccinations with a 21-day interval and were challenged with 2020GD02 at 21 days after the second vaccination. Clinical signs, serological responses, viral shedding, and histopathological changes were used to estimate protective efficacy of the two vaccines. Compared to Group 2, animals in Group 1 produced higher level FHV-1 antibody titers during immune processes. After challenge, Group 3 developed typical FVR. In contrast, animals in both Groups 1 and 2 showed significantly fewer clinical signs, viral shedding, and pathological changes, but could not provide complete protection. Our results provided a reference for further FHV-1 vaccines development in China.

## 1 Introduction

Feline herpesvirus-1 (FHV-1) is a member of the family *Herpesviridae*, subfamily *Alphaherpesvirinae*, genus *Varicellovirus*. It is an enveloped virus with linear, double-stranded DNA, approximately 134 kb in length (1). FHV-1 is a highly prevalent pathogen worldwide, capable of infecting various feline species with a high degree of species specificity. It is a major cause of respiratory and ocular disease in cats, with infection rates reaching as high as 97% in some populations, primarily transmitted by infected cats (2). FHV-1 is transmitted by ocular, nasal, and oral secretions, primarily through direct contact with infected cats. Acutely infected animals are a major source of the virus, and latently infected carrier cats can also shed the virus and infect susceptible cats (1).

Genomic studies indicate that FHV-1 exhibits high conservation compared to other herpesviruses (3). Although different FHV-1 isolates can result in varying degrees of disease severity in cats, sequence and phylogenetic analyses reveal that the genomic differences among these isolates are minimal. Nevertheless, FHV-1 is capable of mutating and evolving into distinct branches, highlighting the growing importance of effective prevention and control measures to manage this virus in cats.

Vaccines are the mainstay of prevention and control of FHV-1. The main commercially available FHV-1 vaccines currently include modified live (ML) and inactivated vaccines (4). Inactivated vaccines are considered biologically safer than ML vaccines due to the absence of the risk of viral reversion to virulence (5). However, both vaccines could not completely protect cats against FHV-1 challenge and protection failure cases after vaccination have been reported (6). It has been reported that vaccine efficacy can be compromised by a mismatch between the vaccine and field virus strain (7).

Prior to the initiation of this study, there were no domestically produced, commercially available FHV-1 vaccines in China that utilized field strains as the antigenic component. Most FHV-1 vaccines commercially available in China are inactivated vaccines, which also include components for the prevention of feline calicivirus (FCV) and feline panleukopenia virus (FPV) in addition to FHV-1 collectively known as the feline viral rhinotracheitis, calicivirus, and panleukopenia (FVRCP) vaccine, However, the FHV-1 component of these vaccines has been reported to be the least effective (8). It has been reported this kind of vaccine used in China could not completely prevent cats against FHV-1 infection of field strains (9), which can relieve clinical signs but do not prevent viral shedding or the development or recurrence of latent states.

An epidemiological survey shows that the positivity rate of FHV-1 in some regions of China has reached 4.61% (10). More effective vaccines are needed to prevent and control FHV-1. In the present study, the protective effect of FVRCP vaccine against the Chinese FHV-1 field strains was estimated, which provided a reference for further FHV-1 vaccines development in China.

## 2 Materials and methods

### 2.1 Cells and virus

Crandell Reese Feline Kidney (CRFK) cells were used for FHV-1 propagation and viral titration. The FHV-1 field strain 2020GD02, originally isolated in 2013 from a clinically affected cat in Guangdong Province, China, was plaque-purified on CRFK cells and stored at −80°C in our laboratory.

### 2.2 Preparation of vaccine

The inactivating agent *β*-propiolactone was mixed with FHV-1 at a ratio of 1:2000 and inactivated at 4°C for 36 h, followed by hydrolysis at 37°C for 2 h. The inactivated virus was then emulsified with Montanide™ GEL 02 adjuvant (SEPPIC, France) at a 10:1 (v/v) ratio under continuous stirring at 4°C to prepare the final vaccine formulation.

### 2.3 Animals and study design

Twelve healthy male Chinese Li Hua Cats, obtained from a professional experimental animal facility in Guangdong, China, with an initial body weight ranging from 800 to 900 grams, were randomly divided into three groups (*n* = 4 each). All cats were weaned at 1 month of age and confirmed to be free of antigens and antibodies against FHV-1, FPV, and FCV prior to the experiment at 2 to 3 months of age, thus avoiding potential interference from maternal antibodies. Each group was kept separate during the experimental process.

Group 1 received the commercial FVRCP vaccine (Fel-O-Vax^®^ PCT, Zoetis) via subcutaneous injection according to the manufacturer’s protocol. Group 2 was immunized with 1 mL of the inactivated experimental vaccine containing 10^6.75^ TCID_50_ of FHV-1 2020GD02, administered subcutaneously. Group 3 served as the negative control, mock-vaccinated with PBS. All cats received two immunizations at a 21-day interval.

At 42 days after the first immunization, all cats were challenged with FHV-1 2020GD02. All cats received an intranasal inoculation of 0.5 mL and an ocular conjunctival inoculation of 0.5 mL of the viral solution, resulting in a total viral dose of 10^6.75^ TCID_50_ of FHV-1 2020GD02. Following inoculation, the cats were monitored daily through clinical examinations and scored for several clinical signs (Supplementary Table 1). The monitored clinical signs included conjunctivitis, blepharospasm, ocular discharge, sneezing, nasal discharge, nasal congestion, and body temperature, consistent with established methodologies (4).

The animal study was approved by the Experimental Animal Ethics Committee of South China Agricultural University no. 2023C055. The study was conducted in accordance with the local legislation and institutional requirements.

### 2.4 ELISA antibody assay

Following the first immunization, blood samples (without anticoagulant) were collected weekly from all cats and the serum was separated. Serum was stored at −20°C until indirect enzyme linked immunosorbent assay (ELISA) was performed. Serum FHV-1 antibody titers in serum were detected by indirect ELISA methods established in our laboratory.

The FHV-1 glycoprotein B recombinant protein was diluted in ELISA binding buffer at 200 μg/mL and then added to a 96-well ELISA plate, sealed, and incubated at 4°C overnight. After overnight binding, plates were washed three times with PBS containing 0.05% (v/v) Tween-20 (PBST), blocked with 2% skimmed milk powder diluted with PBST for 1 h at room temperature, and then washed three times. Cat serums were diluted with PBS at a 1:100 dilution and added to the ELISA plates in triplicate, followed by incubation for 1 h at room temperature. The horseradish peroxidase-conjugated rabbit-anti-cat (Biodragon, Beijing, China) antibody was diluted 1:7,500 in PBS, added to the wells, and incubated for 30 min at room temperature. After another five washes, 3,3′,5,5’-Tetramethylbenzidine Substrate Solution for ELISA was added, and the samples were incubated in the dark at 37°C for 30 min. The reaction was stopped by the addition of 50 μL of stop solution to each well, and the absorbance at 450 nm was measured in a microplate reader.

### 2.5 Detection of viral shedding

To monitor viral shedding, nasal swabs were collected daily post-inoculation and placed in 500 μL PBS. After vortex, the swabs were discarded, and the suspensions were centrifuged (5,000 × g, 10 min, 4°C) to pellet cell debris. The resulting supernatant was stored at −80°C until nucleic acid extraction.

Viral nucleic acids were extracted from the supernatant using the MagPure Pathogen DNA/RNA Kit C (Magen Guangdong, China) following the manufacturer’s instructions. To determine the copy numbers of the genome of FHV-1, the extracted viral nucleic acids were used for quantitative PCR. The quantitative PCR assay utilized forward and reverse primers with the following sequences: 5′-ACTTCGATGAGGAAAAGCTAATGC and 5′-GACGGAGGGCCATGTTAGTG, respectively. The copy number for each sample is expressed as log_10_(copies per μL).

### 2.6 Histopathology

At 14 days post-inoculation, one cat from each group was humanely euthanized. Lung and tracheal tissues were collected from the cats, fixed in 10% formalin solution, and processed for histopathological examination by Shanghai Biotechnology Corporation. Tissue sections were stained with hematoxylin and eosin (H&E).

### 2.7 Statistical analyses

Significant differences in data of each group were analyzed by Student’s t-test using GraphPad Prism software 6. Results are presented as mean ± standard deviations (SDs). The *p*-values derived from the analyses are indicated by asterisk. *p*-values are indicated with asterisks: *p < 0.05, **p < 0.01, and ***p < 0.001. Statistical significance was defined as *p* < 0.05. Non-significant results (*p* ≥ 0.05) were not specially marked.

## 3 Results

### 3.1 Antibody responses

In the present study, 12 healthy 2- to 3-month-old cats were randomly divided into three groups (*n* = 4 each). Serum FHV-1-specific antibody levels were dynamically monitored by indirect ELISA. Following first immunization, both group 1 and group 2 showed successful seroconversion, while group 3 remained antibody-negative prior to challenge. Notably, at all-time points post-immunization (7, 14, 28, and 35 days), group 1 exhibited significantly higher antibody titers than group 2 (*p* < 0.05).

**Figure 1:**
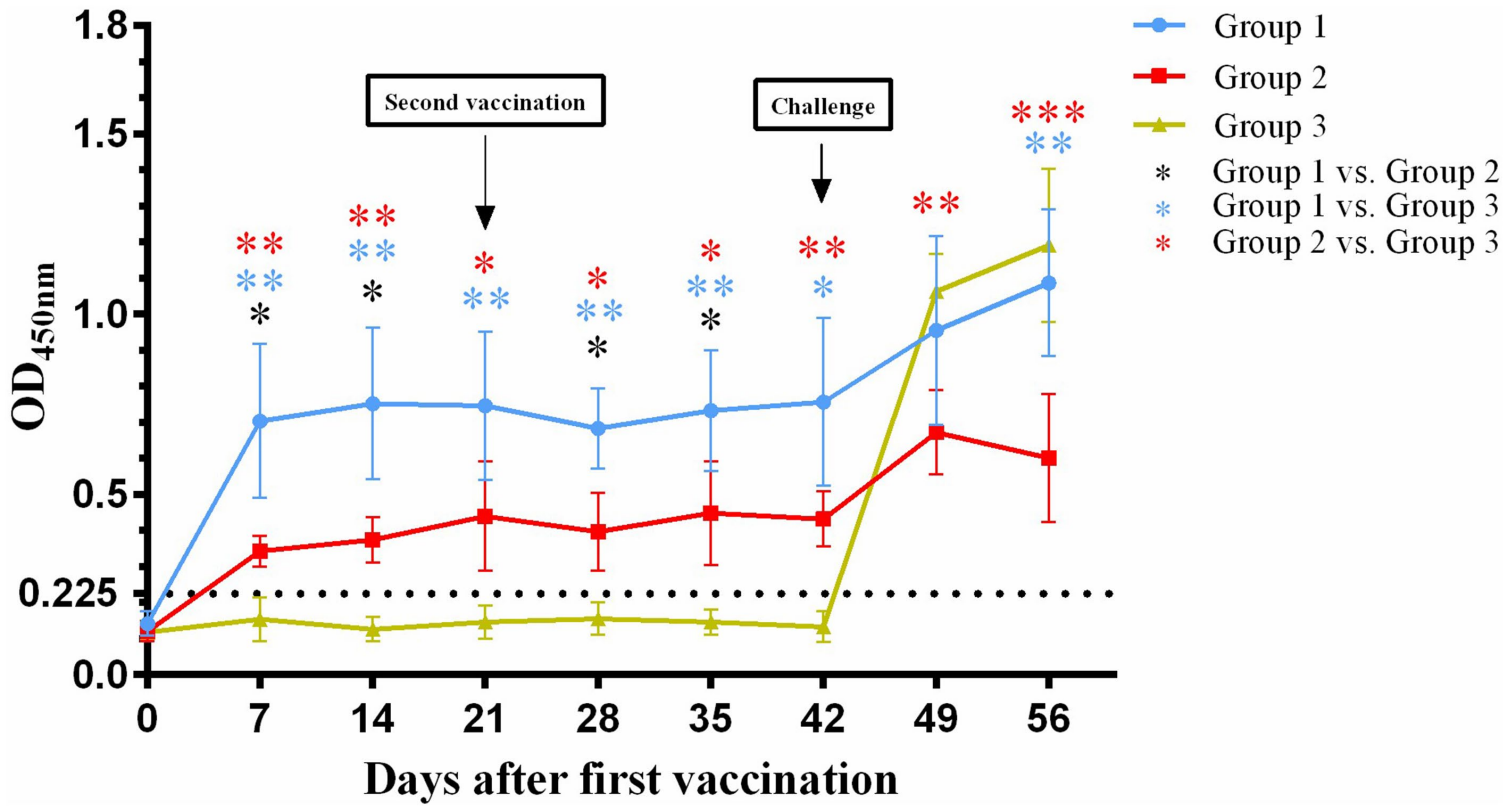
Changes in serum antibody titers against FHV-1after first vaccination (Day 0). The black dotted line represents the detection limit. *p < 0.05; **p < 0.01; ***p < 0.001.

After challenge, both vaccinated groups demonstrated further increases in antibody levels. Group 1 reached peak antibody response at 14 days post-inoculation (OD_450_ = 1.0865), while group 2 showed an earlier response pattern, peaking at 7 days post-inoculation (OD_450_ = 0.6715). Of particular interest, the originally seronegative group 3 also displayed rising antibody titers post-inoculation. This result not only confirmed the reliability of the challenge model but also indicated successful viral infection establishment in all experimental cats. The dynamic characteristics of antibody level changes in each group provided important evidence for evaluating vaccine immunogenicity (Figure 1).

### 3.2 Clinical responses

**Figure 2:**
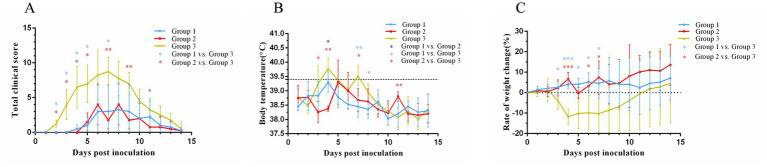
Changes in the clinical score **(A)**, body temperature **(B)**, and weight **(C)** after cats were challenged with FHV-1. The black dotted line **(B)** represents body temperature of 39.4°C. On day 0 after inoculation, the body weights were set as 0 and represented as the black dotted line **(C)**. *p < 0.05; **p < 0.01; ***p < 0.001.

Following virus inoculation, daily monitoring of clinical scores (Figure 2A), body temperature (Figure 2B), and body weight (Figure 2C) was conducted across all groups. In group 1, one cat exhibited mild blepharospasm at days 11 post-inoculation, while two other cats displayed mild clinical signs of sneezing at 4 to 5 days post-inoculation lasting for about 10 days with nasal serous discharge, blepharospasm and mild or moderate nasal congestion. Group 2 showed ocular serous discharge, mild or moderate sneezing, nasal congestion, and nasal serous or mucoid discharge 5 to 8 days post-inoculation, with the most clinically severe cat having nasal discharge that lasted for 4 days. Notably, one cat in each vaccinated group remained completely asymptomatic throughout the observation period. Statistical analysis revealed significantly lower clinical scores in group 1 compared to group 3 at days 2–5 and 7 post-inoculation (*p* < 0.05), while group 2 demonstrated superior protection with significantly reduced scores versus group 3 at days 2–6, 9, and 11 post-inoculation (*p* < 0.05 or *p* < 0.01). In contrast, all cats in group 3 exhibited typical clinical signs of FVR within 2 days post-inoculation, including sneezing, ocular and nasal discharge, nasal congestion, and mild conjunctivitis.

Cats in group 3 started to have fever at 3 days post-inoculation (Figure 2B), and one cat reached a maximum temperature (40.2°C) at 4 days post-inoculation. Compared with cats in group 3, cats in group 1 had a lower maximum body temperature (39.8°C) and a shorter duration of fever; cats in group 2 had a later onset of fever, and body temperature of one cat reached a maximum value of being 40.3°C.

Group 3 exhibited initial weight loss at 3 days post-inoculation (Figure 2C), reaching the lowest point at 4 days post-inoculation with an 11.8% reduction in body weight. This was followed by a gradual increase in body weight. Group 1 showed weight loss at 3 days post-inoculation, followed by weight gain of 6% finally. In contrast, group 2 showed two significant weight losses at 5 days and 8 days post-inoculation, with an overall trend of gradual weight gain, culminating in a 16.7% increase in body weight.

### 3.3 Viral shedding

**Figure 3:**
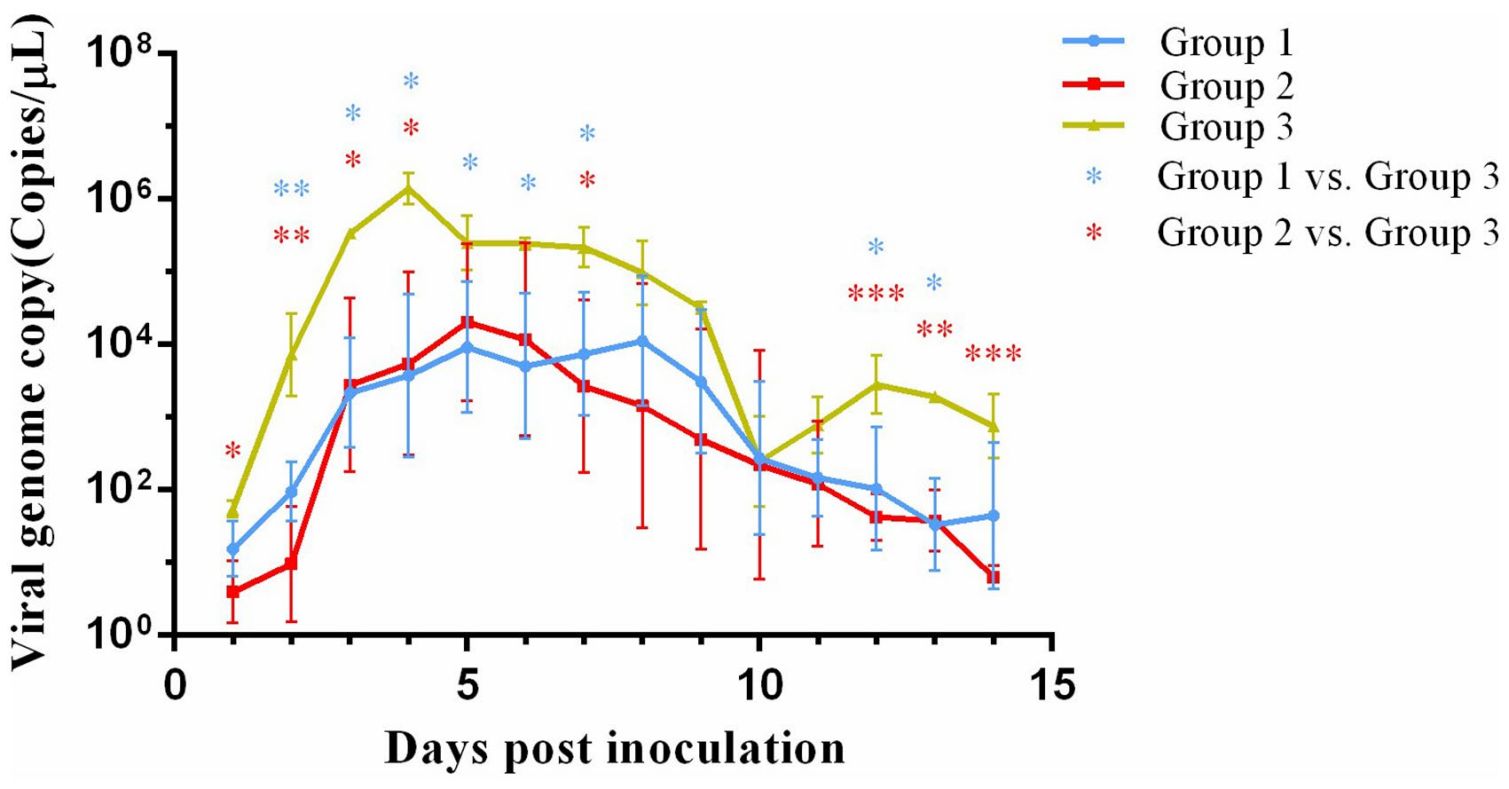
Viral genome load in nasal swab collected from cats after challenge. *p < 0.05; **p < 0.01; ***p < 0.001.

All experimental groups demonstrated detectable viral shedding for 14 days post-inoculation as quantified by qPCR (Figure 3). A significant difference (*p* < 0.01) in viral shedding was detected between control group and both vaccinated groups. In group 3, viral shedding reached its peak (10^6.193^ copies/μL) at 4 days post-inoculation and then declined. A secondary increase in viral shedding was noted in group 3 at 10 days post-inoculation. Notably, the peak levels of viral shedding in both group 1 (10^4.461^ copies/μL) and group 2 (10^4.488^ copies/μL) were lower than those observed in group 3. No significant differences in viral shedding were observed between group 1 and group 2.

### 3.4 Histopathology

**Figure 4:**
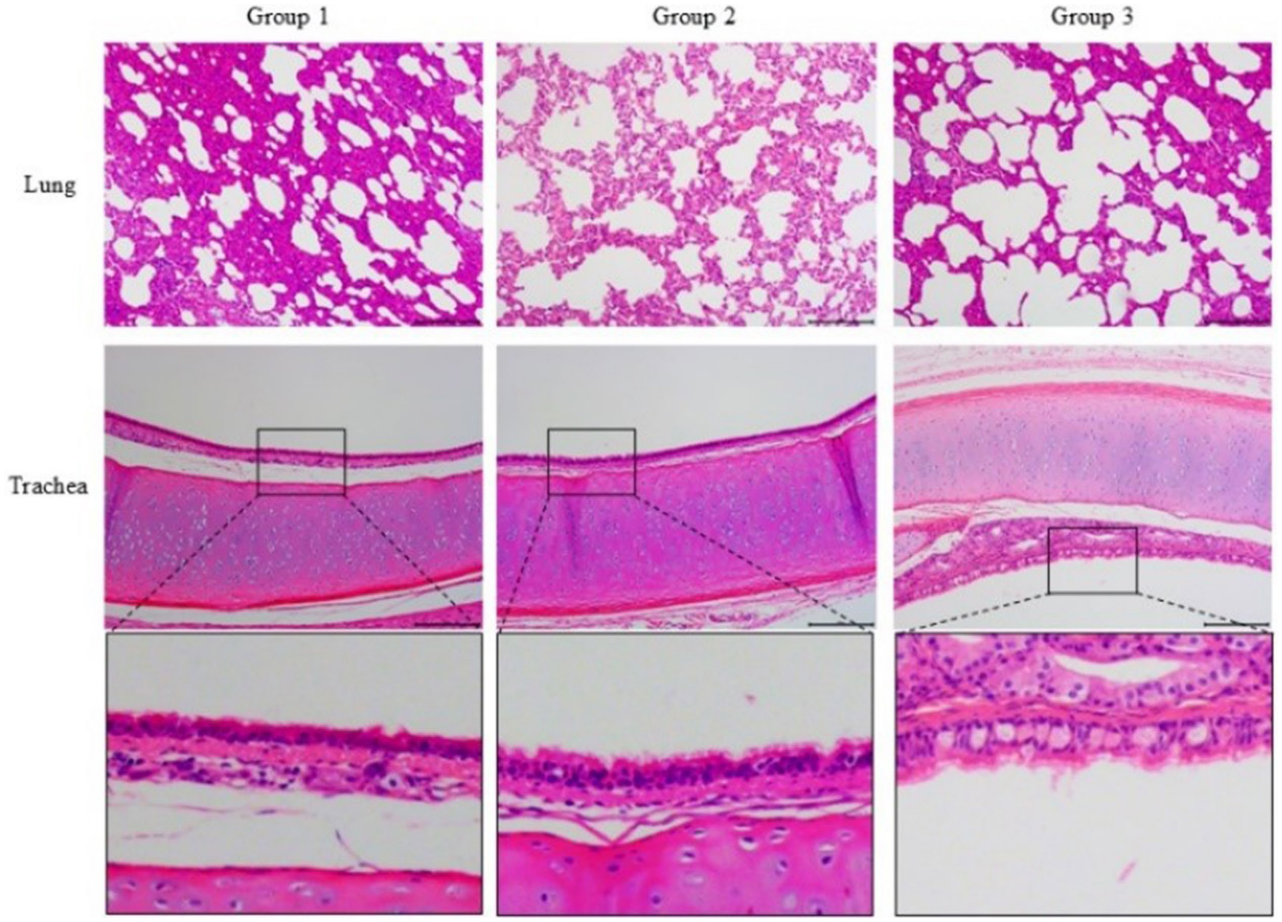
Histopathological sections (H&E, ×100) of the lung and trachea of challenged cats. Group 3 showed severe alveolar septa disruption, inflammatory cell infiltration, and tracheal epithelial desquamation. Groups 1 and 2 exhibited milder damage and reduced inflammation. The third row presents a magnified view of the trachea.

In the cat of group 3 (Figure 4), it was observed a disruption of the alveolar septa, thickening of the alveolar walls, and significant detachment of septal cells, accompanied by an infiltration of inflammatory cells within the alveolar septa. In contrast, the cats in group 1 and group 2 exhibited less severe damage with a reduced presence of inflammatory cells.

Additionally, the tracheal epithelium of the cat in group 3 showed hypertrophy and was infiltrated by inflammatory cells, with the highest degree of epithelial cell desquamation. In comparison, the tracheal epithelium of cats in group 1 and group 2 remained relatively intact, with less epithelial cell shedding.

## 4 Discussion

In the present study, the protective efficacy of the FHV-1 field vaccine and the FVRCP vaccine was estimated through clinical scores, body weight, body temperature, antibody titers, viral shedding and histopathological changes. Both vaccines alleviated FHV-1-induced clinical signs, including fever, weight loss, and upper respiratory symptoms. Notably, the clinical scores of cats vaccinated with the FHV-1 field vaccine were comparable to those that received the FVRCP vaccine. However, the vaccines exhibited differential protection profiles: the FVRCP vaccine provided better control of respiratory symptoms, while the FHV-1 field vaccine showed superior ocular protection. This disparity may be attributed to the vaccine strain. It has also been suggested that this difference in respiratory and ocular symptoms may be related to the sex, breed and immune status of the cats, or to the adjuvant used in the vaccine (4, 11). Therefore, the protective effect of the vaccine against respiratory and ocular symptoms could be researched within these contexts in further research.

Monitoring of body temperature revealed that unvaccinated cats exceeded the normal temperature range within 3 to 5 days post-inoculation, whereas vaccinated cats (both inactivated and FVRCP groups) exhibited transient fever that resolved within 2 days, demonstrating effective mitigation of fever symptoms. However, both vaccines did not eliminate the viral shedding, and only reduced viral shedding in the cats. Although FHV-1 nucleic acid was detected in nasal swabs of all cats, viral shedding was significantly lower in vaccinated cats compared with controls. This reduction in viral shedding is consistent with findings from other studies (12), suggesting that both the inactivated vaccine and the FVRCP vaccine were effective in reducing the spread of FHV-1 in the cat population. During this period, there were cats in both Group 1 and Group 2 that did not show any clinical signs, suggesting that there was no positive correlation between viral shedding and clinical signs, which is consistent with related reports (13).

For viral shedding detection, qPCR was selected due to its high sensitivity and rapid turnaround, which were critical for monitoring early and low-level viral shedding. Although qPCR cannot differentiate between infectious and non-infectious viral particles, its advantages in sensitivity and speed made it the most suitable method for this study. Future studies could combine qPCR with cell culture to provide a more comprehensive assessment of viral shedding, including both the presence and infectivity of the virus.

The ocular-nasal route of virus inoculation was used to mimic natural infection conditions. However, the study did not determine the minimum infective dose (MID) of the field strain 2020GD02, and therefore it is not a completely realistic representation of the natural infection of the animals. It is established in animal experimentation that the protective efficacy of vaccines is closely associated with the clinical exposure dose of the pathogen (14). Consequently, a subsequent phase of the study will focus on optimizing the challenge dose to more accurately simulate the scenario where animals become infected and develop disease at doses approaching the MID of the virus. This refinement is essential for enhancing the study’s relevance to natural infection conditions and for obtaining more precise data on vaccine efficacy.

The antigen content, adjuvants, and production processes of vaccines are significantly influence immune responses (15, 16). It is important to emphasize that the FHV-1 field vaccine and the commercial FVRCP vaccine cannot be made identical in terms of antigen content, adjuvants, and production processes due to differences in formulation and manufacturing methods. Future studies will include controlled comparisons with standardized variables to further evaluate the antigen immunogenicity.

FHV-1 exhibits low recombination rates but undergoes mutation (17, 18). Phylogenetic studies classify global FHV-1 strains into four major geographically distinct branches (18), with several prevalent strains identified in China (19–21). There is evidence to suggest that vaccine efficacy can be significantly compromised when there is a mismatch between the vaccine strain and the prevalent strain (22). Therefore, it is important to estimate the protective efficacy of these vaccines against the more broadly prevalent strains in China to ensure their effectiveness against the currently prevalent FHV-1.

A limitation of this study is the use of a single geographical isolate, which may not fully represent FHV-1’s genetic diversity or cross-protective potential. As a DNA virus, FHV-1 has relatively low mutation rates (17, 18) but future work should incorporate genomic analysis and cross-protection studies with additional isolates to validate broader vaccine applicability.

In summary, immunization and challenge experiments were performed to compare an inactivated vaccine containing the Chinese field strain FHV-1 2020GD02 with a commercial FVRCP vaccine. Both vaccines were effective in relieving clinical symptoms and reducing viral shedding and pathological lesions but could not provide complete protection. This study provided a reference for further FHV-1 vaccines development in China.

## Data Availability

The original contributions presented in the study are included in the article/Supplementary material, further inquiries can be directed to the corresponding authors.
