# Supplementary material for: Protective efficacy of inactivated FHV-1 vaccine in cats following challenge with the Chinese field strains
Source: Front Vet Sci. 2025 Apr 24;12:1571409. doi: 10.3389/fvets.2025.1571409 (PMC12061028; doi:10.3389/fvets.2025.1571409)
Supplement: Supplementary file 1 [file Table_1.docx]

Supplementary Material

# Supplementary Table 1

Clinical scoring system used in the present study after cats were challenged with FHV-1.

| Clinical Sign | Description | Score |
| --- | --- | --- |
| Conjunctivitis | None  Mild conjunctival hyperemia  Moderate to severe conjunctival hyperemia  Moderate to severe conjunctival hyperemia and chemosis | 0  1  2  3 |
| Blepharospasm | None  Eye <25% closed  Eye 25~50% closed  Eye 50~75% closed  Eye completely closed | 0  1  2  3  4 |
| Ocular discharge | None  Minor serous discharge  Moderate mucoid discharge  Marked mucopurulent discharge | 0  1  2  3 |
| Sneezing | None  Observed | 0  1 |
| Nasal discharge | None  Minor serous discharge  Moderate mucoid discharge  Marked mucopurulent discharge | 0  1  2  3 |
| Nasal congestion | None  Minor congestion (barely audible)  Moderate congestion (easily audible)  Marked congestion with open-mouth breathing | 0  1  2  3 |
| Cough | None  Observed | 0  1 |
| Body temperature | ≤39.4℃  ＞39.4℃ | 0  1 |
